# Cell-free synthesis of functional antibody fragments to provide a structural basis for antibody–antigen interaction

**DOI:** 10.1371/journal.pone.0193158

**Published:** 2018-02-20

**Authors:** Takayoshi Matsuda, Takuhiro Ito, Chie Takemoto, Kazushige Katsura, Mariko Ikeda, Motoaki Wakiyama, Mutsuko Kukimoto-Niino, Shigeyuki Yokoyama, Yoshikazu Kurosawa, Mikako Shirouzu

**Affiliations:** 1 Division of Structural and Synthetic Biology, RIKEN Center for Life Science Technologies, 1-7-22 Suehiro-cho, Tsurumiku, Yokohama, Japan; 2 RIKEN Systems and Structural Biology Center, 1-7-22 Suehiro-cho, Tsurumiku, Yokohama, Japan; 3 RIKEN Structural Biology Laboratory, 1-7-22 Suehiro-cho, Tsurumiku, Yokohama, Japan; 4 Innovation Center for Advanced Medicine, Fujita Health University School of Medicine, Toyoake, Aichi, Japan; Duke University School of Medicine, UNITED STATES

## Abstract

Growing numbers of therapeutic antibodies offer excellent treatment strategies for many diseases. Elucidation of the interaction between a potential therapeutic antibody and its target protein by structural analysis reveals the mechanism of action and offers useful information for developing rational antibody designs for improved affinity. Here, we developed a rapid, high-yield cell-free system using dialysis mode to synthesize antibody fragments for the structural analysis of antibody–antigen complexes. Optimal synthesis conditions of fragments (Fv and Fab) of the anti-EGFR antibody 059–152 were rapidly determined in a day by using a 30-μl-scale unit. The concentration of supplemented disulfide isomerase, DsbC, was critical to obtaining soluble antibody fragments. The optimal conditions were directly applicable to a 9-ml-scale reaction, with linear scalable yields of more than 1 mg/ml. Analyses of purified 059-152-Fv and Fab showed that the cell-free synthesized antibody fragments were disulfide-bridged, with antigen binding activity comparable to that of clinical antibodies. Examination of the crystal structure of cell-free synthesized 059-152-Fv in complex with the extracellular domain of human EGFR revealed that the epitope of 059-152-Fv broadly covers the EGF binding surface on domain III, including residues that formed critical hydrogen bonds with EGF (Asp355^EGFR^, Gln384^EGFR^, H409^EGFR^, and Lys465^EGFR^), so that the antibody inhibited EGFR activation. We further demonstrated the application of the cell-free system to site-specific integration of non-natural amino acids for antibody engineering, which would expand the availability of therapeutic antibodies based on structural information and rational design. This cell-free system could be an ideal antibody-fragment production platform for functional and structural analysis of potential therapeutic antibodies and for engineered antibody development.

## Introduction

Antibody therapy offers effective treatment strategies for many diseases with a highly specific binding of a monoclonal antibody to its target antigen protein, when its inactivation leads to the suppression of the disease [1]. Elucidation of the interaction between a potential therapeutic antibody and its target protein is crucial to understand the mechanisms of action of these antibodies and to develop rational antibody design for improved affinity [2]. Epidermal growth factor receptor (EGFR) is one of the major targets of cancer treatment, because increases in the expression and activation levels of EGFR are frequently found in several metastatic cancers [3]. EGFR is composed of three segments: the extracellular domain (ECD), the single membrane-spanning region, and the cytoplasmic tyrosine kinase domain. Epidermal growth factor (EGF) binding to the ECD induces dimerization of EGFR, which leads to activation of the cytoplasmic tyrosine kinase domain and subsequent stimulation of cell growth and differentiation [4]. Many therapeutic antibodies targeting EGFR expressed in tumors are in clinical use or under development. Examination of the crystal structures of anti-EGFR antibodies in complex with EGFR has revealed their detailed mechanisms of inhibition. For example, the antigen-binding fragment (Fab) of cetuximab or IMC-11F8 binds to the EGF-binding site on domain III of EGFR, thereby preventing EGF-induced activation [5, 6]. The matuzumab Fab fragment also interacts with domain III of EGFR, where apart from the EGF-binding site, and it sterically blocks the domain rearrangement of EGFR to the EGF-binding conformation [7]. 059–152 is one of the fully human anti-EGFR antibodies isolated from a human single-chain variable fragment (scFv) phage display library [8]. The 059–152 antibody is a promising candidate for use in EGFR-targeted antibody therapy, because it has potent antitumor activity against A431 epidermoid carcinoma cells. Although 059–152 IgG inhibits the binding of EGF to EGFR as well as the phosphorylation of EGFR, the molecular mechanism is not well understood.

The cell-free protein synthesis system has become a standard protein production method, and it provides several advantages over conventional cell-based expression methods [9, 10]. Because the cell-free system is an open system and free from cell viability, the system offers great flexibility for directly manipulating the reaction environment. For example, supplementing cell-free reactions with the appropriate amount of zinc ion significantly increases the solubility and stability of zinc-binding proteins [11]. Functional membrane proteins can be produced in the presence of the appropriate concentrations of specific detergents and lipids [12]. A dialysis-mode cell-free system can produce milligram quantities of the protein of interest, which is sufficient for many research purposes, including functional and structural analysis [13]. Labeled protein is easily produced by simply replacing natural amino acids with labeled ones, which is useful for structural analysis by X-ray crystallography and nuclear magnetic resonance spectroscopy [14–16]. Site-specific labeling of a protein with non-natural amino acids is accomplished by supplementing with template DNA containing UAG codons at the desired positions, aminoacyl-tRNA synthetase, UAG-reading tRNA, and non-natural amino acid [17, 18].

A Fab is frequently used for the structural analysis of antibody–antigen complexes by X-ray crystallography, because the rigid and uniform structure of the Fab is beneficial for crystal packing [19]. Mammalian cells are usually used to express IgG or Fabs, because eukaryotic chaperones and other folding catalysts are involved in facilitating their folding. A Fab is prepared by enzymatic digestion of IgG by papain, which is expressed from hybridoma cells or transient expression in mammalian cells. However, the optimized parameters for small-scale expression of IgG or Fabs often do not scale up linearly, and in such cases time-consuming re-optimization is required [20]. In addition, inefficient digestion of some IgGs often hinders Fab preparation. Bacterial-cell-based expression systems are routinely used for scFv and Fab preparation and are still advantageous in that they are affordable and enable easy manipulation, although the production of correctly folded eukaryotic proteins is often challenging, particularly when the protein contains multiple disulfide bonds [21, 22]. The flexibility of the cell-free system, which allows direct manipulation of the reaction environment, makes the system suitable for the synthesis of disulfide-containing proteins. By controlling the oxidizing environment to promote appropriate disulfide bond formation, the cell-free system can reportedly be used to synthesize pharmaceutical disulfide-containing proteins, including antibody fragments, without the need for a laborious refolding process [23–26]. Although several groups have reported cell-free synthesis of antibody fragments or IgG, application of the cell-free system to structural analysis by X-ray crystallography has not yet been demonstrated [27–29]. This remains challenging, because X-ray crystallography requires milligram quantities of structurally homogeneous antibody fragments to obtain well-diffracted crystals through crystallization screening.

Here, we developed a method of practical and rapid antibody fragment synthesis for structural analysis. We show that a dialysis-mode cell-free system based on *Escherichia coli* (*E*. *coli*) S30 extract is a powerful tool for producing antibody fragments for structural analysis, including by X-ray crystallography. We used the anti-EGFR antibody 059–152 to demonstrate that milligram quantities of functional Fvs and Fabs can be synthesized. Furthermore, we successfully determined the crystal structure of the 059–152 Fv in complex with human EGFR-ECD, which revealed a novel manner of interaction between anti-EGFR antibody and EGFR and provided the structural basis for EGFR inhibition by 059–152 antibody. As far as we know, this is the first evidence that a cell-free system can be used to synthesize functional, fully human antibody fragments and that cell-free synthesized antibody fragments can be used for sample preparation for X-ray crystallography. Moreover, we demonstrate that the site-specific fluorescent labeling of 059–152 antibody fragments is possible without impairing antigen binding activity.

## Materials and methods

### Plasmid construction

The genes encoding the VHCH1 and light chains of 059-152-antibody were synthesized by a gene synthesis service (Eurofins Operon, Germany) with codon usage optimization for bacterial expression. The nucleotide sequences encoding the 059-152-VH and the 059–152 light chain (LC) have been deposited in GenBank (accession numbers LC307162 and AB064177.1, respectively). The nucleotide sequence encoding the CH1 domain of human IgG1 was used for that encoding VHCH1, in accordance with the literature [8]. We subcloned the genes encoding 059-152-VH, 059-152-VL, 059-152-VHCH1, and the 059-152-LC into the pCR2.1 vector by using a two-step PCR method. The DNA sequences encoding the T7 promoter, ribosome-binding site, N11-tag, and tobacco etch virus (TEV) protease recognition site were attached at the 5′-end of the coding sequence, whereas that for the T7 terminator was attached at the 3′-end [30]. Note that signal peptide was excluded from the coding sequence. The resulting plasmids were named pCR2.1-059-152-VH, pCR2.1-059-152-VL, pCR2.1-059-152-VHCH1, and pCR2.1-059-152-LC.

### Cell-free synthesis of 059-152-Fv and Fab

The dialysis mode of cell-free synthesis was used to synthesize 059-152-Fv and Fab. A small-scale dialysis unit (30 μl internal and 300 μl external solution) was used to optimize the reaction conditions [11]. The composition of the internal solution was 60 mM HEPES-KOH buffer (pH 7.5) containing 200 mM potassium L-glutamate, 15 mM magnesium acetate, 1.5 mM each amino acid, 1.3 mM ATP, 0.9 mM GTP, 0.9 mM CTP, 0.9 mM UTP, 81 μM folinic acid, 27.6 mM ammonium acetate, 80 mM creatine phosphate, 1 mM reduced glutathione (GSH), 4 mM oxidized glutathione (GSSG), 175 μg/ml tRNAs, 0.2 to 0.8 mg/ml disulfide isomerase (DsbC), 100 μg/ml creatine kinase, 60 μg/ml T7 RNA polymerase, 30% (v/v) *E*. *coli* S30 extract, and 2 μg/ml each template DNA. To produce the Fv, pCR2.1-059-152-VH and pCR2.1-059-152-VL were added to the internal solution. The plasmids pCR2.1-059-152-VHCH1 and pCR2.1-059-152-LC were added to produce the Fab. Variable concentrations of *E*. *coli* DsbC were added to the internal solution to determine its optimal concentration. The external solution was composed of the same components as the internal solution except that DsbC, creatine kinase, T7 RNA polymerase, and template DNAs were omitted. In addition, dithiothreitol-free S30 buffer was used instead of S30 extract [26]. The cell-free reaction was performed at 25°C for 7 h, unless otherwise noted.

### Preparation of 059-152-Fv and Fab for X-ray crystallography

Large-scale preparation of 059-152-Fv was performed as follows. 059-152-Fv was synthesized by using a large-scale dialysis unit (9 ml internal and 90 ml external solution, hereafter referred to as 9–90 ml scale) in the presence of 1 mM GSH, 4 mM GSSG, and 0.2 mg/ml DsbC. After the cell-free synthes is reaction, the internal solution was diluted with four times the volume of buffer A [20 mM Tris-HCl buffer (pH 7.5) containing 500 mM NaCl and 20 mM imidazole] and then centrifuged at 4,500 × *g* for 5 min. After being passed through a 0.45-μm filter unit, the supernatant was subjected to affinity chromatography using a HisTrap HP column (GE Healthcare, USA), and the 059-152-Fv was eluted with a linear gradient of 0% to 100% buffer B [20 mM Tris-HCl buffer (pH 7.5) containing 500 mM NaCl and 500 mM imidazole]. The eluted fractions were pooled and dialyzed against buffer A by using a membrane with a molecular weight cutoff (MWCO) of 3.5 kDa. To cleave the N11 tag, 20 μg/ml TEV protease was added to the solution, which was then incubated at 30°C for 3 h. The cleaved tag was removed by passing the solution through a HisTrap HP column. The flow-through fraction, containing 059-152-Fv, was collected and concentrated by using a centrifugal filter unit with a MWCO of 3.5 kDa (Millipore, USA). 059-152-Fv was further purified by size exclusion chromatography (SEC) using a Hiload 16/60 Superdex 200 column equilibrated with buffer C [20 mM Tris-HCl buffer (pH 7.5) containing 150 mM NaCl]. The concentration of purified 059-152-Fv was determined by UV absorbance at 280 nm. 059-152-Fab was synthesized in the presence of 1 mM GSH, 4 mM GSSG, and 0.8 mg/ml DsbC. 059-152-Fab was purified by using the same procedure as for 059-152-Fv, with minor modifications: a dialysis membrane and a centrifugal filter unit with a MWCO of 10 kDa were used.

### Preparation of EGFR-ECD

C-terminally His-tagged EGFR-ECD (1–643) was transiently overexpressed in HEK293S GnTI-cells [31]. The secreted C-terminally His-tagged EGFR-ECD was recovered from the medium by being passed through a HisTrap HP column. After the column had been washed with buffer A, the EGFR-ECD was eluted with a linear gradient of 0% to 100% buffer B. The eluted fractions were dialyzed against buffer A. The His-tag was then cleaved by TEV protease and removed by a HisTrap HP column. The N-linked oligosaccharides of the EGFR-ECD were cleaved by endoglycosidase H treatment. The EGFR-ECD was buffer-exchanged by dialysis into 20 mM Tris-HCl buffer (pH 8.5) containing 10 mM NaCl. The N-linked oligosaccharides cleaved EGFR-ECD was separated from the EGFR-ECD with the remaining extra oligosaccharide(s) on an anion exchange column (HiTrap Q HP) by using a linear gradient of 0% to 25% solution of 20 mM Tris-HCl buffer (pH 8.5) containing 500 mM NaCl. The main peak was concentrated by using a centrifugal filter unit and purified by SEC using a Hiload 16/60 Superdex 200 column equilibrated with buffer C. The concentration of purified EGFR-ECD was determined by UV absorbance at 280 nm.

To prepare complexes of EGFR-ECD•059-152-Fv and EGFR-ECD•059-152-Fab for crystallization, the antibody fragments were mixed with EGFR-ECD at a molar ratio of 1.2:1. The complex was separated from excess antibody fragments by using the SEC column equilibrated with 20 mM Tris-HCl buffer (pH 8.5) containing 150 mM NaCl. Fractions containing the complex were confirmed by SDS-PAGE and were then concentrated to 15 mg/ml by using a centrifugal filter unit.

### Crystallization, data collection, and refinement

Crystals of the 059-152-Fv•EGFR-ECD complex were obtained by using the sitting drop vapor diffusion method from a drop containing equal volumes of 059-152-Fv•EGFR-ECD solution and a reservoir solution [0.1 M MES buffer (pH 6.0) containing 0.1 M Zn(OAc)_2_ and 6% (w/v) PEG8000]. The crystals were immersed stepwise in reservoir solutions containing increasing concentrations of glycerol [15%, 20%, 25%, and 30% (v/v)]. The crystals were then flash cooled in liquid nitrogen. X-ray diffraction data were collected at the Photon Factory beamline AR-NE3A (Ibaraki, Japan). The dataset was processed by using HKL-2000 software [32]. The initial phase was determined with molecular replacement method using the software Phaser [33]. As a search model, the crystal structures of the extracellular domain of human EGFR-ECD [Protein Data Bank (PDB) ID code: 3NJP] and the Fv region of anti-EGFR Fab (PDB ID code: 3B2U) were used. The model was refined with the software Coot and Phenix [34, 35].

### Kinetic analysis

Binding analysis was performed with a BIAcore 3000 (GE Healthcare). The EGFR-ECD was immobilized on a CM5 sensor chip by using an amine coupling kit (GE Healthcare). Kinetic analyses were performed using single-cycle kinetic analysis by measuring five different concentration series (50.0, 16.6, 5.55, 1.85, and 0.617 nM) of 059-152-Fv or 059-152-Fab in each cycle. Sensorgrams were obtained at a flow rate of 30 μl/min, with a 60-s association phase and a 120-s dissociation phase at 25°C in HBS-EP+ buffer. After each cycle, the sensor surface was regenerated with an injection of 10 mM glycine-HCl (pH 2.4). Kinetic parameters were determined with BIAevaluation software (GE Healthcare). All kinetic data are the results of triplicate experiments.

### Preparation of site-specific fluorescent-labeled antibody fragments

We used inverse PCR to insert the DNA sequence encoding the GSSGSS linker and an amber codon into the plasmids pCR2.1-059-152-VH and pCR2.1-059-152-VHCH1 just before the stop codon to produce pCR2.1-059-152-VH-GSSGSS-amber and pCR2.1-059-152-VHCH1-GSSGSS-amber. To synthesize site-specific p-azido-L-phenylalanine (AzF)-incorporated antibody fragments, 9–90 ml scale cell-free reaction was performed in the presence of 0.2 mg/ml AzFRS (orthogonal aminoacyl-tRNA synthetase specific for AzF), 2 mM AzF, and S30 extract prepared from *E*. *coli* strain B-95.ΔA harboring the UAG-reading tRNA-expressing plasmid pACYC184-piodoTyrRS-MJR1 [36]. For the synthesis of C-terminally AzF-incorporated Fv, the template DNAs of pCR2.1-059-152-VH-GSSGSS-amber and pCR2.1-059-152-VL were added to the internal solution, whereas pCR2.1-059-152-VHCH1-GSSGSS-amber and pCR2.1-059-152-LC were used to synthesize AzF-incorporated Fab. AzF-incorporated Fv and Fab were purified as described above. S30 extract of strain B-95.ΔA was prepared as described in the literature [37].

AzF-incorporated 059-152-Fv and Fab were site-specifically fluorescent labeled with Alexa-488 DIBO alkyne (Thermo Fisher Scientific, USA) by using copper-free click chemistry. Using a rotator, 20 μM antibody fragments were gently mixed with 200 μM Alexa-488 DIBO alkyne for 16 h at room temperature in PBS buffer. Alexa-488 conjugated Fv and Fab were isolated from the residual fluorescent dye in a PD-10 column equilibrated with PBS buffer. Labeling efficiency and protein concentration were evaluated by using the protein and label method of NanoDrop spectrophotometers (Thermo Fisher Scientific). Fluorescence of the site-specifically Alexa-488-conjugated 059-152-Fv and 059-152-Fab was confirmed by reducing and non-reducing SDS-PAGE, as follows. Homogeneously purified 0.5 μg aliquots of each of 059-152-Fv, AzF-incorporated 059-152-Fv, and Alexa-488-conjugated 059-152-Fv, as well as 059-152-Fab, AzF-incorporated 059-152-Fab, and Alexa-488-conjugated 059-152-Fab, was resolved on 10% to 20% gradient gels. Fluorescent gel images were acquired by using the fluorescence method (excitation wavelength, 460 nm; emission filter, Y515) of an ImageQuant LAS-4000 (GE Healthcare). The same gels were then stained with Coomassie Brilliant Blue (CBB), and images were obtained by using the digitizing method of the LAS-4000.

## Results

### Cell-free synthesis of 059-152-Fv and 059-152-Fab

**Fig 1:**
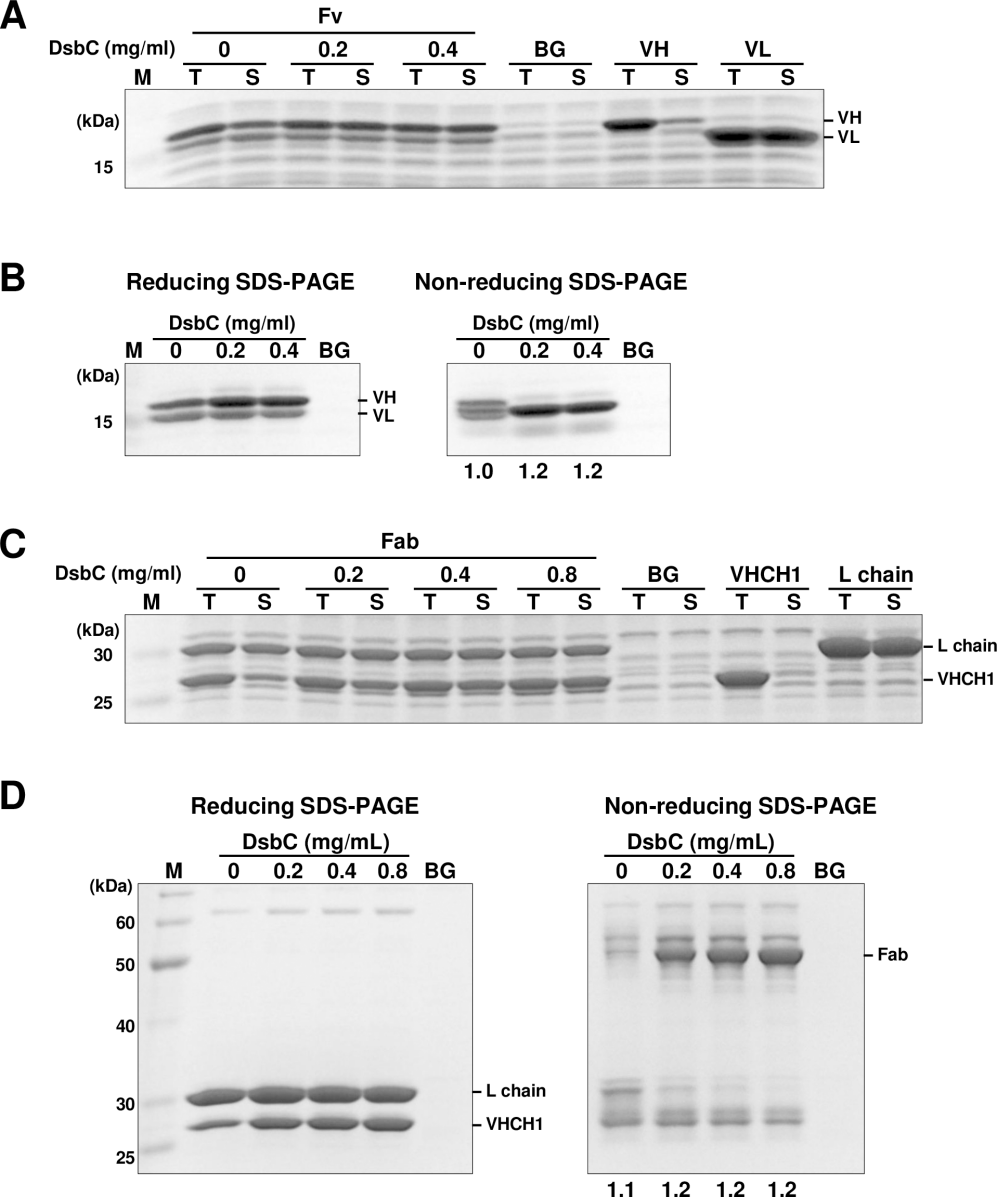
SDS-PAGE analysis of cell-free synthesized 059-152-Fv and 059-152-Fab. (A) 059-152-Fv was synthesized under a series of different concentrations (0, 0.2, and 0.4 mg/ml) of DsbC, as indicated. Total (T) and soluble (S) fractions of the internal solution were analyzed by reducing SDS-PAGE. (B) Purified 059-152-Fv was analyzed by reducing and non-reducing SDS-PAGE. The yields (mg per 1 ml internal solution) of partially purified Fv are indicated under each lane of the non-reducing SDS polyacrylamide gel image. (C) 059-152-Fab was synthesized in the presence of 0, 0.2, 0.4, and 0.8 mg/ml of DsbC, as indicated. (D) Purified 059-152-Fab was analyzed by reducing and non-reducing SDS-PAGE. The yields (mg per 1 ml internal solution) of partially purified Fab are indicated under each lane of the non-reducing SDS polyacrylamide gel image. BG: cell-free synthesis without template DNA. VH: cell-free synthesis of VH without DsbC. VL: cell-free synthesis of VL without DsbC. VHCH1: cell-free synthesis of VHCH1 without DsbC. L chain: cell-free synthesis of light chain without DsbC. Gels were stained with CBB.

A Fv contains two pairs of disulfide bonds, and a Fab contains five pairs. In many extracellular proteins, disulfide bond formation and folding are closely coupled processes. We therefore co-translationally facilitated appropriate disulfide bond formation for the synthesis of functional antibody fragments. Cell-free synthesis was performed under precisely controlled oxidative conditions in the presence of 1 mM GSH and 4 mM GSSG to promote disulfide bond formation. In addition, bacterial disulfide isomerase, DsbC was introduced to the cell-free reaction to reshuffle incorrectly formed disulfide bonds. 059-152-Fv was synthesized in the presence of three different concentrations of DsbC (0, 0.2, or 0.4 mg/ml). 059-152-VH and 059-152-VL were mostly soluble under all three conditions. The solubility of 059-152-VH was increased slightly by adding more than 0.2 mg/ml DsbC (Fig 1A). When synthesized without DsbC, partially purified 059-152-Fv showed several bands on non-reducing SDS-PAGE (Fig 1B). This was likely to have been 059-152-Fv that contained incorrectly formed disulfide bond(s) and was thus inactive. Therefore, to obtain correctly disulfide-bonded 059-152-Fv, DsbC needed to be added at a concentration of at least 0.2 mg/ml. Next, 059-152-Fab was synthesized under four different concentration series of DsbC (0, 0.2, 0.4, or 0.8 mg/ml). Whereas the solubility of the light chain was independent of supplementation with DsbC, the solubility of the VHCH1 chain was strongly dependent on the DsbC concentration (Fig 1C). The maximum yield of soluble Fab was obtained when DsbC was supplemented at 0.8 mg/ml, as judged from the band in non-reducing SDS-PAGE of partially purified Fab (Fig 1D). Formation of inter-chain disulfide bond between the VHCH1 chain and the light chain was confirmed by comparing the reducing and non-reducing SDS-PAGE images. Therefore, we determined the ideal synthesis conditions for Fv and Fab (DsbC at 0.2 mg/ml for Fv and 0.8 mg/ml for Fab), based on their band patterns and intensities on the non-reducing SDS polyacrylamide gel, as well as the yields of the soluble products. Under ideal conditions, approximately 1 mg of Fv or Fab was successfully synthesized from 1 ml of internal solution. Interestingly, in contrast with the cell-free syntheses of VL and light chain alone, which were soluble in the absence of DsbC, the VH and VHCH1 chains alone formed insoluble aggregates (Fig 1A and 1C). This indicated that reshuffling of incorrect disulfide bonds by DsbC was an essential step for correct folding of the VH and VHCH1 chains. Considering our findings that solely synthesized VHCH1 still tended to aggregate even when supplemented with a sufficient amount of DsbC and that co-expression of the light chain considerably improved the solubility of VHCH1 (S1 Fig and Fig 1C), we surmised that the light chain had chaperone-like activity to facilitate productive folding of VHCH1.

### Kinetic analysis

We prepared purified 059-152-Fv and 059-152-Fab from 9–90 ml scale cell-free synthesis. The yields were 10.8 mg and 14.5 mg, respectively. Kinetic analysis was performed by surface plasmon resonance to verify the binding activity of the cell-free synthesized 059-152-Fv and 059-152-Fab to immobilized EGFR-ECD. 059-152-Fv and 059-152-Fab bound to the EGFR-ECD with *K*_D_ values of 5.9 nM and 7.7 nM, respectively (Table 1). These *K*_D_ values are comparable to those of the Fabs of the pharmaceutical anti-EGFR antibodies cetuximab (2.3 nM) and IMC-11F8 (3.3 nM) [5]. The k_on_ and k_off_ values (Table 1), as well as the sensorgrams (S2 Fig), indicated that 059-152-Fv and 059-152-Fab had the characteristics of fast binding and dissociation. These analyses showed that the cell-free system was capable of synthesizing functional antibody fragments.

**Table 1:** Binding kinetics of 059-152-Fv and 059-152-Fab to the EGFR-ECD.

	*k*_on_ (10^5^ M^-1^s^-1^)	*k*_off_ (10^−3^ s^-1^)	*K*_D_ (nM)
059-152-Fv	24.9 ± 0.8	14.7 ± 0.5	5.9 ± 0.1
059-152-Fv/AzF	23.6 ± 0.1	13.7 ± 0.4	5.8 ± 0.2
059-152-Fv/Alexa-488	25.2 ± 0.6	15.4 ± 0.2	6.1 ± 0.1
059-152-Fab	19.4 ± 0.5	15.0 ± 0.1	7.7 ± 0.1
059-152-Fab/AzF	17.8 ± 0.4	12.5 ± 0.4	7.0 ± 0.1
059-152-Fab/Alexa-488	16.7 ± 0.5	13.3 ± 0.7	8.1 ± 0.2

Association rate (*k*_on_), dissociation rate (*k*_off_), and binding affinity (*K*_D_) of 059-152-Fv, 059-152-Fab, AzF-incorporated Fv, AzF-incorporated Fab, Alexa-488 conjugated Fv, and Alexa-488 conjugated Fab to EGFR-ECD are shown.

### Site-specific fluorescent-labeled antibody fragment production

Genetic incorporation of non-natural amino acids has a wide variety of applications in protein engineering. B-95.ΔA is an *E*. *coli* strain in which the UAG codon is assigned to a non-natural amino acid [36]. This strain is constructed by replacing 95 of 273 UAG codons with one of the other two stop codons (UAA or UGA) in the *E*. *coli* BL21 (DE3) genome. Then the *prfA* gene, which encodes release factor 1, is deleted from the genome to assign the UAG codon to a non-natural amino acid. B-95.ΔA shows active growth comparable to that of the parental *E*. *coli* BL21 (DE3) and is thus suitable for recombinant protein production. We prepared S30 extract from B-95.ΔA, harboring a UAG-reading-suppressor tRNA expression plasmid, and used it for site-specific incorporation of AzF into 059-152-Fv and 059-152-Fab in the cell-free system. The C-terminal of 059-152-VH and 059-152-VHCH1 was chosen as a suitable incorporation site, because the C-terminal region of VH and VHCH1 is distant from the antigen binding site and is less likely to interfere with the folding process of VH and VHCH1. The DNA sequence encoding the GSSGSS linker was inserted before the AzF incorporation site to keep AzF slightly away from the antibody fragments, which would enhance the labeling efficiency.

**Fig 2:**
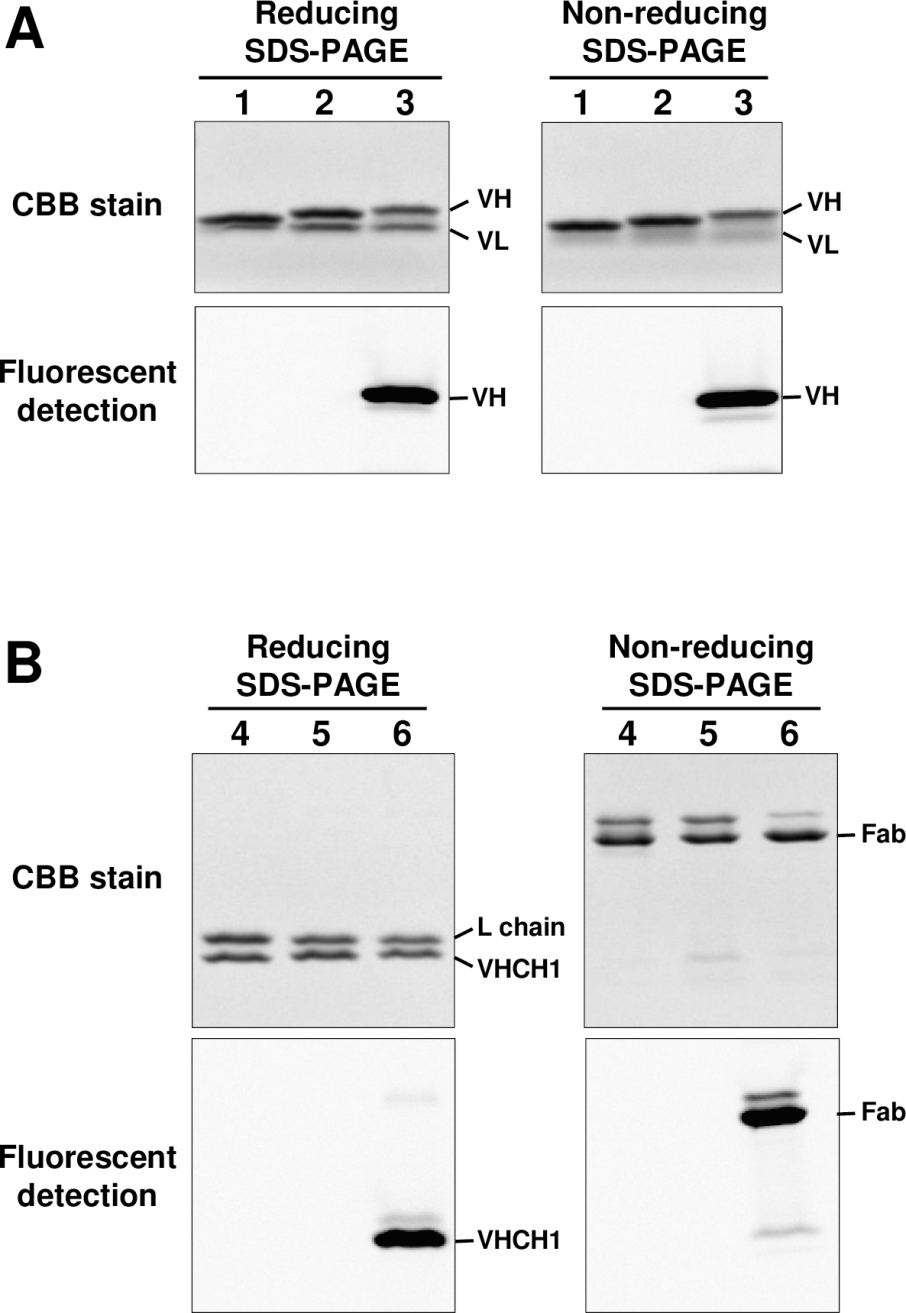
SDS-PAGE analysis of site-specific fluorescent-labeled 059-152-Fv and 059-152-Fab. Reducing and non-reducing SDS-PAGE analysis of 059-152-Fv (A) and 059-152-Fab (B). (lane 1) 059-152-Fv, (lane 2) AzF-incorporated 059-152-Fv, (lane 3) Alexa-488 conjugated 059-152-Fv, (lane 4) 059-152-Fab, (lane 5) AzF-incorporated 059-152-Fab, and (lane 6) Alexa-488 conjugated 059-152-Fab. Fluorescent images and CBB-stained images were acquired from the same gels.

AzF-incorporated 059-152-Fv and 059-152-Fab were successfully synthesized in the cell-free system supplemented with 0.2 mg/ml AzFRS and 2 mM AzF. The yields of homogeneously purified AzF-containing 059-152-Fv and 059-152-Fab from the 9–90 ml-scale cell-free reactions were 12.5 mg and 7.6 mg, respectively. We applied strain-promoted azide-alkyne cycloaddition, a bio-orthogonal chemical reaction, to the fluorescent dye–antibody conjugation reaction. After removing the residual fluorescent dye, we measured the labeling efficiency and it was 76% for 059-152-Fv and 87.9% for 059-152-Fab. Reduced and non-reduced SDS-PAGE analysis clearly showed the fluorescence of the Alexa-488-conjugated 059-152-Fv and 059-152-Fab (Fig 2). Notably, in the reduced SDS polyacrylamide gel image of 059-152-Fv (Fig 2A, lane 1), the VH and VL bands were close together and looked like a single band. In the non-reduced SDS-PAGE image we found a faint band that migrated slightly slower than the Fab. This was presumably due to partial reduction of the Fab during SDS-PAGE. Kinetic analysis revealed that the AzF-incorporated and Alexa 488-conjugated 059-152-Fv and 059-152-Fab retained their native binding affinities to the antigen (Table 1).

### Determination of the structure of the 059-152-Fv•EGFR-ECD complex

The 059-152-Fv•EGFR-ECD complex was prepared by mixing 059-152-Fv and EGFR-ECD at a molar ratio of 1.2:1, it was then isolated by size-exclusion chromatography. High-quality crystals were obtained with a reservoir composed of 0.1 M MES buffer (pH 6.0) containing 0.1 M Zn(OAc)_2_ and 6% (w/v) PEG8000, and diffracted up to 2.9 Å. The initial phase was determined by molecular replacement using the coordinates of the extracellular domain of EGFR (PDB ID code: 3NJP) and the Fv region of anti-EGFR Fab antibody (PDB ID code: 3B2U) as search models. The asymmetric unit contained one 059-152-Fv•EGFR-ECD complex, and the structure was refined up to 2.9 Å. The statistics of data collection and refinement are shown in Table 2.

**Table 2:** Data collection and refinement statistics.

Data collection and refinement	059–152 Fv•EGFR-ECD complex
Data collection	
Beam line	BL-NE3A in PF
Space group	P6_5_22
Cell dimensions	
a, b, c (Å)	203.4, 203.4, 113.8
α, β, γ (°)	90, 90, 120
Resolution (Å)	50.00–2.9 (3.0–2.9)
R_sym_	0.098 (2.053)
*I*/*σI*	39.8 (1.8)
Redundancy	21.7 (22.1)
Completeness (%)	99.6 (100.0)
Refinement	
Resolution (Å)	32.5–2.89
No. of reflections	30595
*R*_work_/*R*_free_	0.2768/0.3309
Number of atoms	6623
B-factors (Å^2^)	94.5
R.m.s. deviations	
Bond length (Å)	0.003
Bond angles (°)	0.560
Ramachandran plot	
Favored/allowed (%)	93.8/6.3
PDB ID code	5XWD

Values in parentheses are for the highest-resolution shell.

### Overall structure of the 059-152-Fv•EGFR-ECD complex

**Fig 3:**
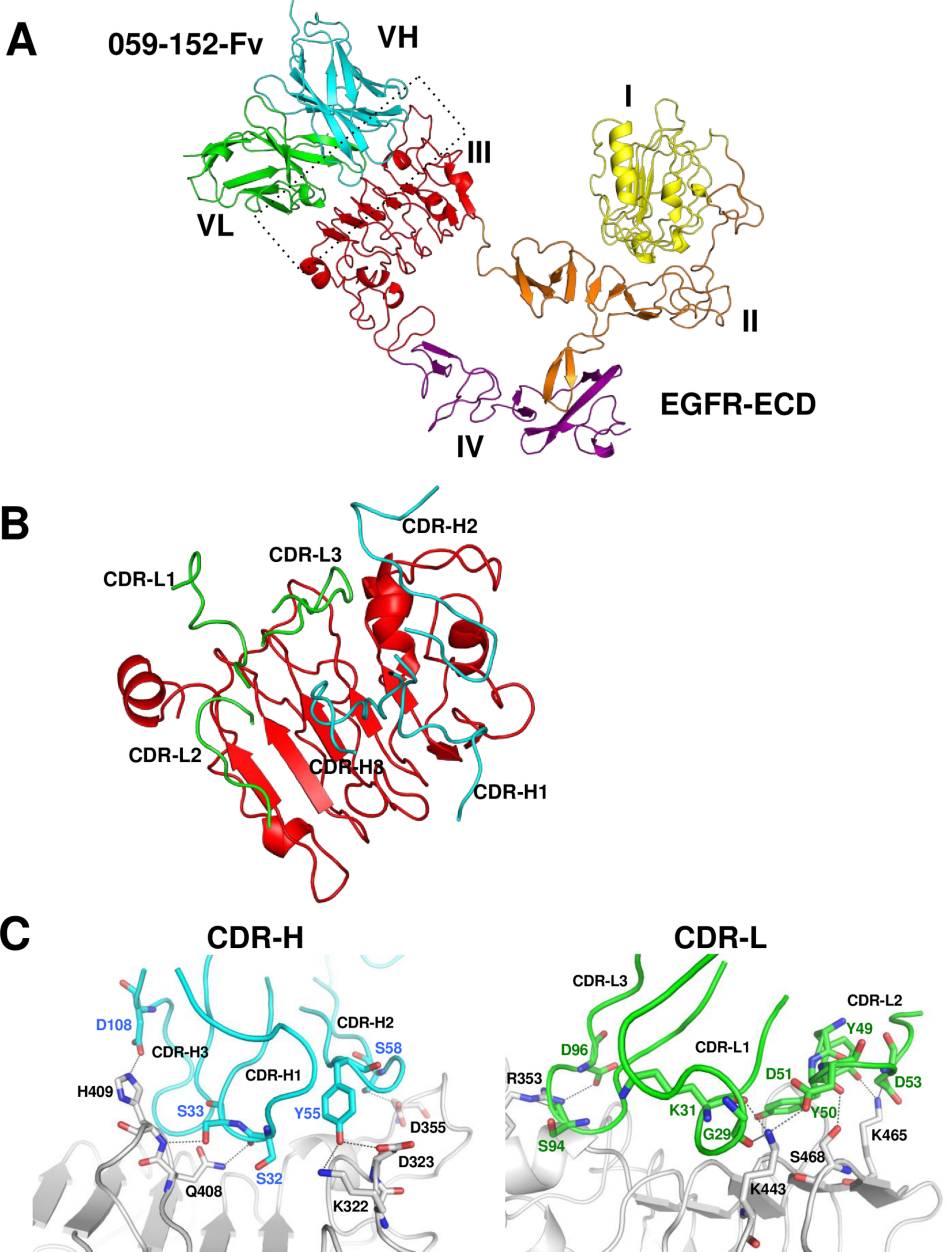
Crystal structure of the 059-152-Fv•EGFR-ECD complex. (A) Ribbon representation of the 059-152-Fv•EGFR-ECD complex. VH and VL domains of 059-152-Fv are respectively colored cyan and green. External region of the EGFR is shown with domain I in yellow, domain II in orange, domain III in red, and domain IV in purple. (B, C) Close up-view of the interactions between CDR loops of 059–152 and domain III. For clarity, interactions of CDR-H and CDR-L are separately shown. CDR-H loops, CDR-L loops, and domain III are colored cyan, green, and gray, respectively. Residues that make key interactions are shown in the stick models. Hydrogen bonds are indicated by gray dotted lines.

**Fig 4:**
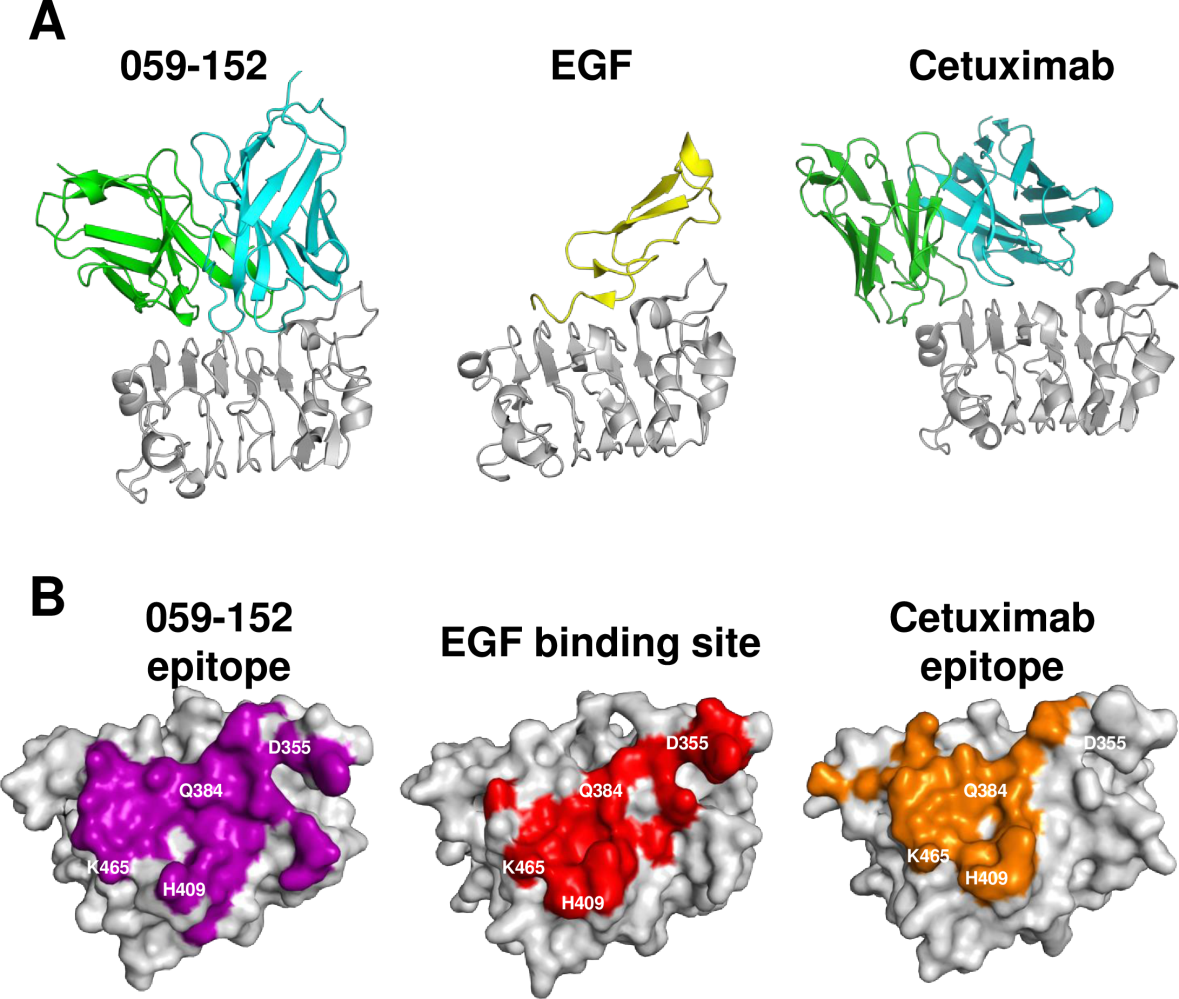
Contacting region of 059-152-Fv, cetuximab, and EGF on domain III. (A) Ribbon representations of domain III in complex with 059-152-Fv, EGF, and cetuximab. VH, VL, EGF, and domain III are respectively shown in cyan, green, yellow, and gray. (B) Surface representations of domain III with the contact region. Surface of domain III (gray) contacting (within 4 Å) 059–152, EGF, and cetuximab are shown in purple, red, and orange, respectively. These are viewed from approximately the same orientations onto the domain III binding site. Residues on domain III that form hydrogen bonds with EGF are shown in white.

**Fig 5:**
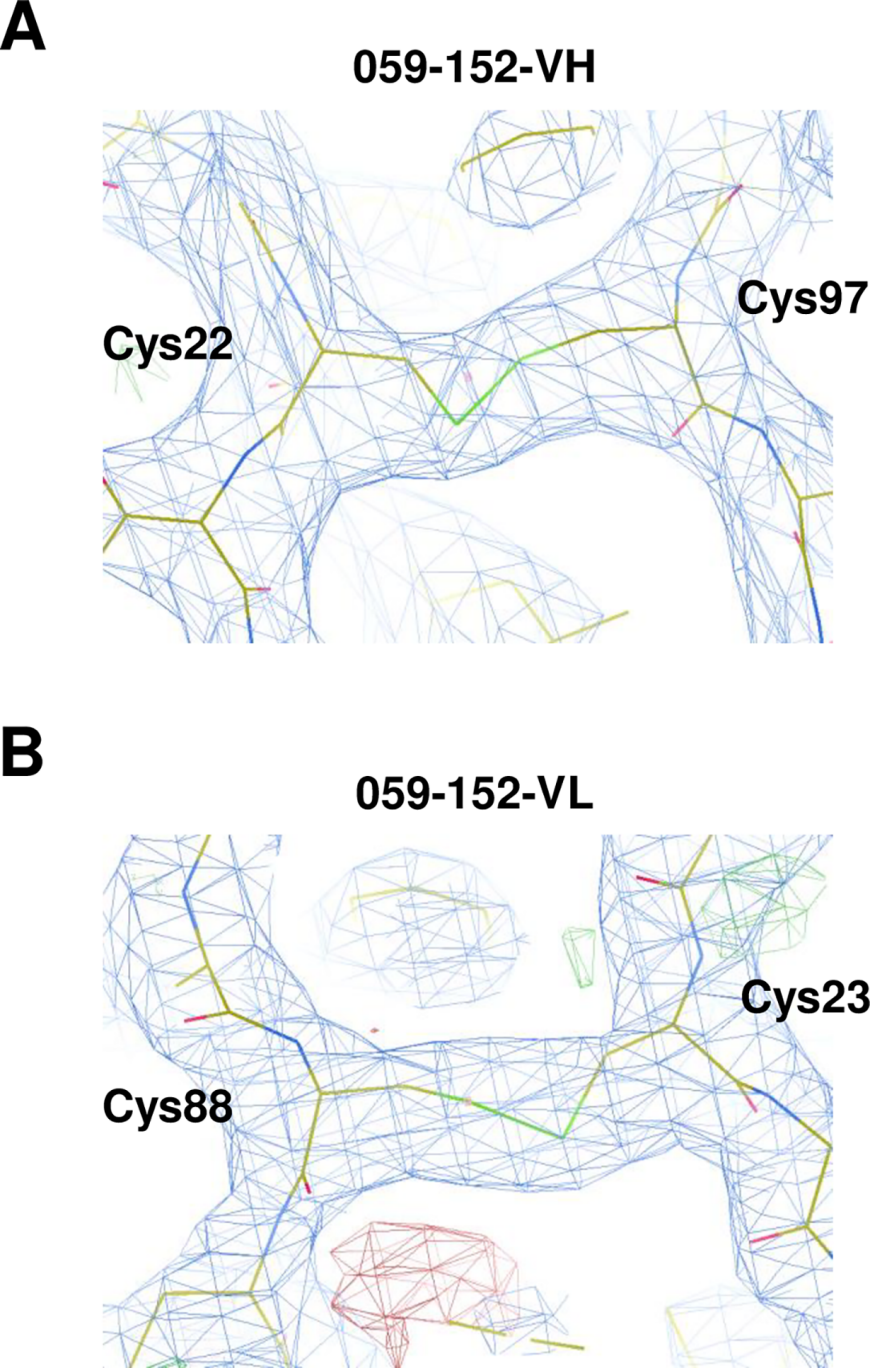
Electron density map of intradomain disulfide bonds of 059-152-Fv. Close-up view of the electron density maps (blue mesh) and stick models of 059-152-VH (A) and 059-152-VL (B) are shown. The stick models are shown with carbons colored yellow; nitrogens, blue; oxygens, red; and sulfurs, green.

Examination of the crystal structure of the 059-152-Fv•EGFR-ECD complex revealed that 059-152-Fv binds exclusively to domain III of the EGFR in a different manner from those of the previously determined structures of antibody•EGFR-ECD complexes (Fig 3A and S3 Fig). The binding site of 059-152-Fv on EGFR domain III largely overlaps with that of EGF (Fig 4), which is consistent with previous information that 059–152 IgG competes with EGF for binding to the EGFR [8]. The domain arrangement of EGFR-ECD is similar to the previously reported monomeric tethered conformation, which is characterized by intermolecular interaction between the dimerization interface of domain II and the protruding β-sheet-like loop in domain IV [5]. Note that the electron densities of native intradomain disulfide bonds were clearly observed between Cys22^VH^ and Cys97^VH^, and between Cys23^VL^ and Cys88^VL^, providing evidence that the cell-free system can synthesize antibody fragments containing natively paired disulfide bonds (Fig 5).

We superimposed the overall EGFR-ECD structure on that of the cetuximab•EGFR-ECD complex by using the coordinates from the PDB ID code, 1YY9. We found that the proximity and relative orientation of the four domains of the EGFR-ECD are similar to those of the latter complex (S3A and S3B Fig). Superimposition of the structures of domain III from the EGFR-ECD complexes with those of 059-152-Fv, cetuximab Fab, and EGF revealed that the root-mean-square-deviation values of domain III between the 059-152-Fv and cetuximab complexes and between the 059-152-Fv and EGF complexes are 0.613 Å and 0.738 Å, respectively. In accordance with this striking similarity, the overall conformation of domain III does not alter upon binding to the antibodies or the ligand factor EGF.

### Interaction between 059-152-Fv and domain III of the EGFR-ECD

All complementarity-determining regions (CDRs) of 059-152-Fv are involved in specific interactions with domain III of EGFR-ECD (Fig 3B and 3C). Basically, shape-complementary hydrophobic interactions between the Fv and domain III are supported by many specific intermolecular hydrogen bonds. CDR-H1 interacts with the loop from Thr406 to Phe412 of domain III through two hydrogen bonds between the main-chain CO group of Ser32^VH^ and the side-chain NH_2_ group of Gln408^EGFR^ and between the side-chain hydroxyl group of Ser33^VH^ and the main-chain NH group of His409^EGFR^. CDR-H2 is located near the N-terminal part of domain III and recognizes two loops of domain III; the side-chain hydroxyl group of Tyr55^VH^ forms hydrogen bonds with both the side-chain amino group of Lys322^EGFR^ and the carboxyl group of Asp323^EGFR^, and the side-chain hydroxyl group of Ser58^VH^ interacts with the side-chain carboxyl group of Asp355 ^EGFR^. In CDR-H3, the side-chain carboxyl group of Asp108^VH^ hydrogen bonds with the side chain of His409 ^EGFR^. The main-chain CO groups of Gly29^VL^ and Lys31^VL^ in CDR-L1 and the side-chain carboxyl group of Asp51^VL^ in CDR-L2 cooperatively recognize the side-chain amino group of Lys443^EGFR^. As for the other residues in CDR-L2, the main-chain CO group of Tyr50^VL^ hydrogen bonds with the side-chain hydroxyl group of Ser468^EGFR^, and the side-chain hydroxyl group of Tyr49^VL^ and the side-chain carboxyl group of Asp53^VL^ interact with the side-chain amino group of Lys465^EGFR^. The main-chain CO group of Ser94^VL^ and the side-chain carboxyl group of Asp96^VL^ in CDR-L3 interact with the side-chain guanidino group of Arg353^EGFR^.

### Comparison of binding sites on EGFR domain III

We compared the binding surfaces of domain III in complex with 059-152-Fv, EGF, and cetuximab Fab to show how these binding sites overlap (Fig 4A and 4B). The epitope of 059-152-Fv broadly covers the EGF binding surface on domain III, including residues that formed critical hydrogen bonds with EGF (Asp355^EGFR^, Gln384^EGFR^, H409^EGFR^, and Lys465^EGFR^). Compared with 059-152-Fv, the epitope of cetuximab is located near the C-terminal part of domain III and similarly prevents hydrogen bond formation of EGF with Gln384^EGFR^, H409^EGFR^, and Lys465^EGFR^. An apparent difference is that cetuximab is positioned distantly from Asp355^EGFR^, whereas 059-152-Fv is located close to it.

### Mechanism of inhibition of EGFR by 059–152 antibody

As described above, a large part of the epitope of 059–152 overlaps with that of cetuximab. Although 059–152 covers a broader area of domain III and is located near the N-terminal part of domain III, 059–152 is likely to exert its inhibition through a mechanism similar to that of cetuximab. We assume that, as is the case with cetuximab, 059–152 antibody binding to domain III sterically blocks the access of ligands to the ligand-binding region on domain III of EGFR, so that 059–152 antibody prevents ligand-induced dimerization [5].

## Discussion

Here, we developed a practical and rapid method of producing functional antibody fragments by using an *E*. *coli* S30-based dialysis-mode cell-free system, and we used it to elucidate the structural basis of EGFR-ECD recognition by the 059–152 antibody. Structural analysis of 059-152-Fv•EGFR-ECD revealed in detail the mechanism of inhibition of the receptor by the 059–152 antibody. 059–152 binds specifically to domain III of EGFR and blocks ligand binding as well as domain rearrangement from an inactive tethered monomer to a ligand high-affinity conformation [38]. We have provided new evidence that human antibody fragments produced by using the cell-free system can be available for structural analysis by X-ray crystallography. Unlike with time-consuming bacterial expression systems, we can prepare partially purified antibody fragments in a day, because the cell-free system can be used to synthesize approximately 1 mg of Fv or Fab from 1 ml internal solution in 7 h and the product is subjected directly to affinity purification steps without cell disruption. Although our cell-free system uses bacterial cell extracts, supplementation with sufficient DsbC in an oxidative environment was the key to synthesizing functional Fvs and Fabs. Recently, the use of antibody fragments has been shown to facilitate the crystallization of membrane proteins by expanding the hydrophilic interface that is beneficial to increase protein–protein interaction in the crystal lattice [39]. Our cell-free system can be applied to this application—a further demonstration of the system’s usefulness [40].

Fabs are frequently used for structural analysis in complex with an antigen, because the Fab can be readily prepared by IgG digestion with papain and subsequent single-step purification using a protein-A affinity column. In this study, unfortunately, we could not obtain well-diffracting crystals of the 059-152-Fab•EGFR-ECD complex. Generally, it is difficult to determine why the target sample did not ideally crystallize without obtaining its crystal structure. However, when using the 059-152-Fab, the packing contacts in the currently determined crystal of the 059-152-Fv•EGFR-ECD complex are not properly formed, because of the steric hindrance caused by the extra CH1 and CL domains. Therefore, crystallization with different forms of the antibody fragments would be an effective choice to obtain well-diffracting crystals. For this purpose, our cell-free system is a rapid and useful single platform for producing Fvs and Fabs in parallel.

Conventionally, the non-site-specific conjugation method, which involves lysine or cysteine residues, is commonly used to label antibody fragments [41, 42]. However, the heterogeneity of the prepared conjugate (in terms of different stoichiometries and conjugation sites) has some disadvantages. One of the foreseeable difficulties is that if fluorescent dye is conjugated to lysine residues in the antigen-binding site, the resulting conjugate might lose the specific affinity for the antigen. Fluorescent dye conjugation should not distort the native tertiary structure of the antibody fragment or impair the fragment’s function. Our novel conjugation method will enable the rational design of homogeneous fluorescent dye–antibody conjugates, which are favorable in terms of the stability, specificity, and affinity of the conjugates. The method should be applicable to several imaging modalities, including immuno-positron emission tomography, fluorescence imaging, and single photon emission computed tomography.

Because of its speed and suitability for parallel protein expression, the cell-free system can be a valuable tool in antibody engineering for screening mutants from mutant libraries [43]. Due to their fast dissociation rates, 059-152-Fv or 059-152-Fab would be suitable for use in affinity engineering models. For example, guided by structural information, introducing amino acid substitutions to the interface of 059–152 to gain shape complementary would be a promising approach to improving affinity. The potential of this approach would be further enhanced if it were combined with expanded genetic code technology that enables efficient and multiple-site integration of synthetic amino acids in addition to the 20 natural amino acids. The incorporation of synthetic amino acids reportedly enhanced the structural stability of enzymes [44], and it would be interesting to apply a similar approach to improve the property, function, and utility of antibodies. Our cell-free system would be a suitable sample preparation platform for this purpose.

## Supporting information

S1 Fig Cell-free synthesis of 059-152-VH and 059-152-VHCH1. (A) 059-152-VH was synthesized under different concentrations of DsbC (0, 0.2, and 0.4 mg/ml), as indicated. (B) 059-152-VHCH1 was synthesized in the presence of 0, 0.2, 0.4, and 0.8 mg/ml of DsbC, as indicated. Total (T) and soluble (S) fractions (0.4 μl) of internal solution were analyzed by reducing SDS-PAGE. VH: cell-free synthesis of 059-152-VH. VHCH1: cell-free synthesis of 059-152-VHCH1. BG (background): cell-free synthesis without template DNA.
(PDF)

S2 Fig Single kinetic analyses of 059–152 antibody fragments. Sensorgrams representative of triplicate measurements are shown. A, 059-152-Fv; B, 059-152-Fv/AzF; C, 059-152/Alexa-488; D, 059-152-Fab; E, 059-152-Fab/AzF; F, 059-152-Fab/Alexa-488. For each analysis, the experimental sensorgrams (black lines) were overlaid with the theoretical fitted curves (colored lines).
(PDF)

S3 Fig Overall structures of anti-EGFR antibody in complex with EGFR-ECD or domain III. Ribbon representation of the structures of anti-EGFR antibody fragments in complex with EGFR-ECD or with domain III are shown. A: 059-152-Fv•EGFR-ECD. B: cetuximab Fab•EGFR-ECD (PDB ID code: 1YY9). C: GC1118 Fab•EGFR-ECD (PDB ID code: 4UV7). D: IMC-11F8 Fab•domain III (PDB ID code: 3B2U). E: matuzumab Fab•domain III (PDB ID code: 3C09). The VH and the VHCH1 chains are colored cyan. The VL and light chains are colored green. The EGFR-ECD is shown with domain I in yellow, domain II in orange, domain III in red, and domain IV in purple. These structures are viewed from approximately the same orientations, using domain III as a standard. The matuzumab Fab•domain III complex is also shown, in 90º rotated view for ease of viewing.
(PDF)
