# Supplementary figures and images for: Cell-free synthesis of functional antibody fragments to provide a structural basis for antibody–antigen interaction
Source: PLoS One. 2018 Feb 20;13(2):e0193158. doi: 10.1371/journal.pone.0193158 (PMC5819829; doi:10.1371/journal.pone.0193158)

**A**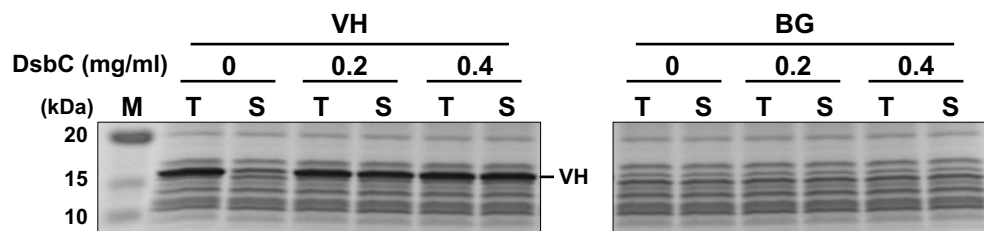**B**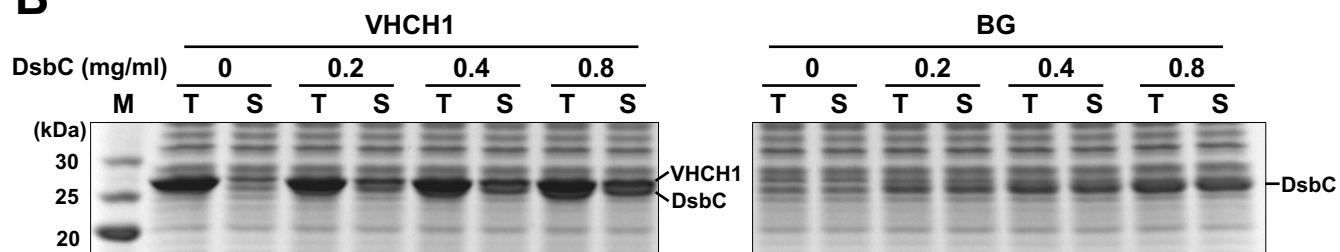

**S1 Fig. Cell-free synthesis of 059-152-VH and 059-152-VHCH1**

Supplement: S1 Fig — (A) 059-152-VH was synthesized under different concentrations of DsbC (0, 0.2, and 0.4 mg/ml), as indicated. (B) 059-152-VHCH1 was synthesized in the presence of 0, 0.2, 0.4, and 0.8 mg/ml of DsbC, as indicated. Total (T) and soluble (S) fractions (0.4 μl) of internal solution were analyzed by reducing SDS-PAGE. VH: cell-free synthesis of 059-152-VH. VHCH1: cell-free synthesis of 059-152-VHCH1. BG (background): cell-free synthesis without template DNA. (PDF) [file pone.0193158.s001.pdf]

**A**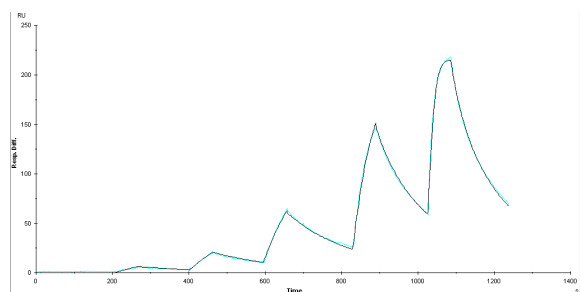**D**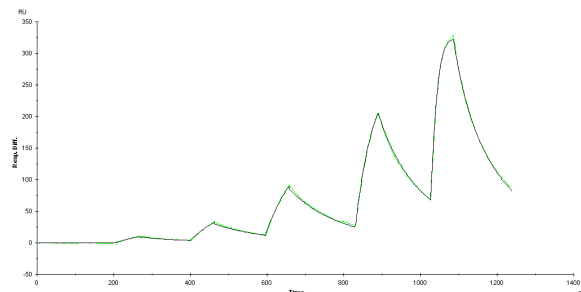**B**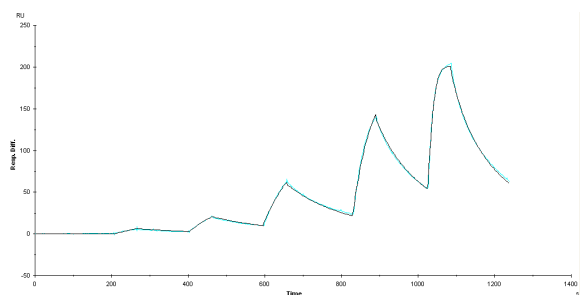**E**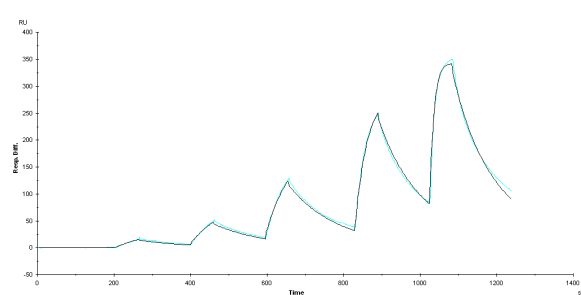**C**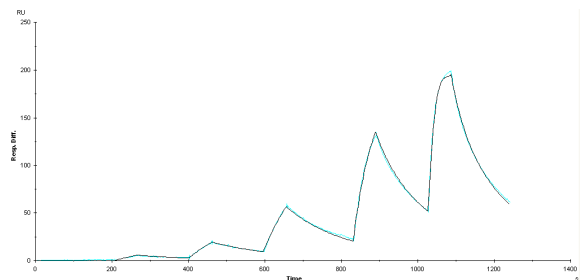**F**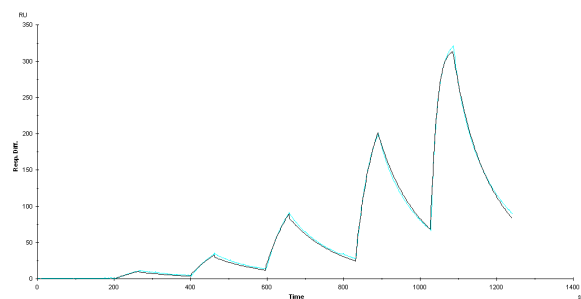

**S2 Fig. Single kinetic analyses of 059-152 antibody fragments**

Supplement: S2 Fig — Sensorgrams representative of triplicate measurements are shown. A, 059-152-Fv; B, 059-152-Fv/AzF; C, 059-152/Alexa-488; D, 059-152-Fab; E, 059-152-Fab/AzF; F, 059-152-Fab/Alexa-488. For each analysis, the experimental sensorgrams (black lines) were overlaid with the theoretical fitted curves (colored lines). (PDF) [file pone.0193158.s002.pdf]

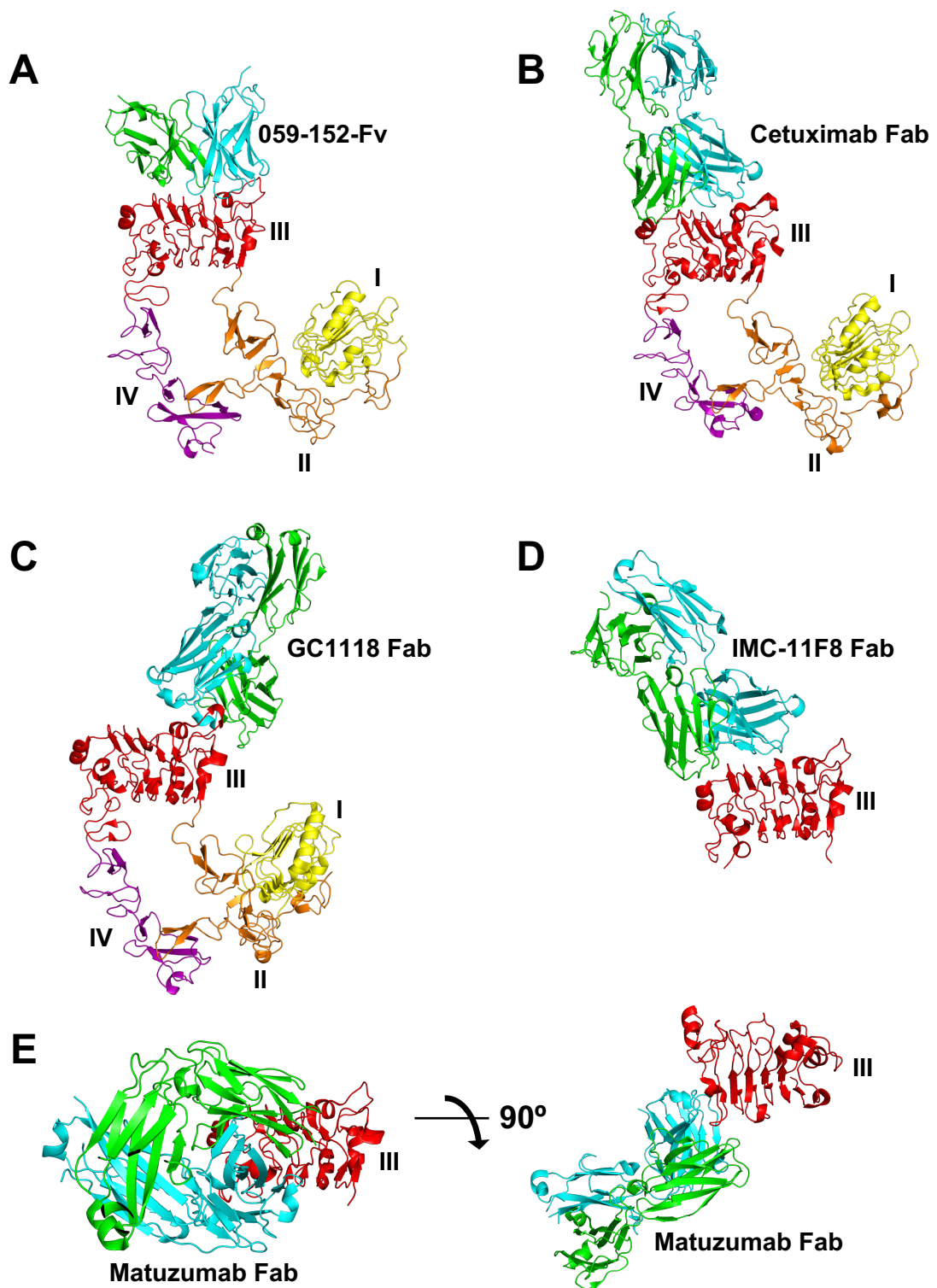

**S3 Fig. Overall structures of anti-EGFR antibody in complex with EGFR-ECD or domain III**

Supplement: S3 Fig — Ribbon representation of the structures of anti-EGFR antibody fragments in complex with EGFR-ECD or with domain III are shown. A: 059-152-Fv•EGFR-ECD. B: cetuximab Fab•EGFR-ECD (PDB ID code: 1YY9). C: GC1118 Fab•EGFR-ECD (PDB ID code: 4UV7). D: IMC-11F8 Fab•domain III (PDB ID code: 3B2U). E: matuzumab Fab•domain III (PDB ID code: 3C09). The VH and the VHCH1 chains are colored cyan. The VL and light chains are colored green. The EGFR-ECD is shown with domain I in yellow, domain II in orange, domain III in red, and domain IV in purple. These structures are viewed from approximately the same orientations, using domain III as a standard. The matuzumab Fab•domain III complex is also shown, in 90º rotated view for ease of viewing. (PDF) [file pone.0193158.s003.pdf]
